# New Appliances & Things Medical

**Published:** 1911-04-01

**Authors:** 


### NEW APPLIANCES & THINGS MEDICAL.

### BUXTON TABLE WATER.

In a former issue we gave particulars regarding the composition of the waters of Buxton Spa (see The Hospital, March 25, p. 739). The bottled water, samples of which have been sent to us, is remarkably pure, and compares favourably with any water of a similar class on the market. As a table water it is to be preferred to those waters which contain a larger amount of iron, since the chalybeate taste is almost absent, while the small amount of saline material it contains gives in the gas impregnated water just that pleasant sharpness of flavour which is so much relished in a good sparkling table water. In the samples submitted to us no impurities were detected, showing that the statement that every care is taken in the bottling is no idle boast, but a precaution, which can be relied upon. The Buxton table water is really of such a high quality that we are surprised to find that it is so comparatively little known. It is a water that ought to be procurable in every restaurant and refreshment bar, and we are convinced that once it has been effectively tried it will be found to be wholly-deserving of the highest praise that can be given to a table water of its kind.

